# How to create more supportive supervision for primary healthcare: lessons from Ngamiland district of Botswana: co-operative inquiry group

**DOI:** 10.3402/gha.v9.31263

**Published:** 2016-06-24

**Authors:** Oathokwa Nkomazana, Robert Mash, Silvia Wojczewski, Ruth Kutalek, Nthabiseng Phaladze

**Affiliations:** 1 Faculty of Medicine, University of Botswana, Gaborone, Botswana; 2 Division of Family Medicine and Primary Care, Stellenbosch University, Stellenbosch, South Africa; 3 Unit Ethnomedicine and International Health, Department of General Practice and Family Medicine, Centre of Public Health, Medical University of Vienna, Vienna, Austria; 4 School of Nursing, University of Botswana, Gaborone, Botswana

**Keywords:** supportive supervision, district health managers, healthcare workers, primary healthcare, co-operative inquiry group

## Abstract

**Background:**

Supportive supervision is a way to foster performance, productivity, motivation, and retention of health workforce. Nevertheless there is a dearth of evidence of the impact and acceptability of supportive supervision in low- and middle-income countries. This article describes a participatory process of transforming the supervisory practice of district health managers to create a supportive environment for primary healthcare workers.

**Objective:**

The objective of the study was to explore how district health managers can change their practice to create a more supportive environment for primary healthcare providers.

**Design:**

A facilitated co-operative inquiry group (CIG) was formed with Ngamiland health district managers. CIG belongs to the participatory action research paradigm and is characterised by a cyclic process of observation, reflection, planning, and action. The CIG went through three cycles between March 2013 and March 2014.

**Results:**

Twelve district health managers participated in the inquiry group. The major insights and learning that emerged from the inquiry process included inadequate supervisory practice, perceptions of healthcare workers’ experiences, change in the managers’ supervision paradigm, recognition of the supervisors’ inadequate supervisory skills, and barriers to supportive supervision. Finally, the group developed a 10-point consensus on what they had learnt regarding supportive supervision.

**Conclusion:**

Ngamiland health district managers have come to appreciate the value of supportive supervision and changed their management style to be more supportive of their subordinates. They also developed a consensus on supportive supervision that could be adapted for use nationally. Supportive supervision should be prioritised at all levels of the health system, and it should be adequately resourced.

## Introduction

Primary healthcare makes an important contribution to achieving equitable access to comprehensive and cost-effective health services (1). Effective primary healthcare services in many low- and-middle-income countries, however, have been hampered by severe shortages and inequitable distribution of the health workforce (2–5). Migration of health workers has exacerbated the shortage and inequity in many of these countries (3, 4, 6).

Multiple innovative strategies have been tried to increase the number of healthcare workers and to distribute and retain them, especially in rural and remote areas (7, 8). These innovations comprised educational, fiscal, and regulatory approaches as well as diverse incentives and have had varying degrees of success (5, 7–10). Based on existing evidence of effective retention strategies, Buykx et al. (11) developed a framework to guide retention interventions. While the emphasis was on ‘bundling’ multiple strategies for efficacy, the framework underscores the centrality of effective management, collegial support, and supervision.

Botswana has a documented shortage of healthcare workers which is worse in primary care (2). The scarcity of primary healthcare workers has been attributed to inequitable distribution favouring towns and hospital care, an increased need for health workers because of the HIV pandemic, and high attrition (2, 12). Poor working environments and living conditions as well as inadequate human resource management practices, including poor support and supervision, are the main contributors to poor health worker motivation and high attrition (12). The supervision was perceived to be either authoritarian or inadequate and incompetent (12, 13).

Supportive supervision has been shown to improve health workers’ satisfaction, motivation, performance, retention, and may also enhance competence and patient outcomes (14–16). However, implementation of supportive supervision can be hindered by the entrenched authoritarian supervisory paradigm (14). A shift to supportive supervision demands a significant paradigm shift, which supervisors may not always find socially or even culturally acceptable (14, 17). In addition, there is a dearth of successful supportive supervisory practices in low- and middle-income countries to use as benchmarks (15). The sociocultural barriers to supportive supervision may be accentuated in Botswana where many of the healthcare workers have migrated from other African countries (2). This article reports on the findings of participatory action research whose aim was to change the management and supervisory practice of the district health management team (DHMT) and cluster heads in order to discover how to create a more supportive environment for primary healthcare providers. Because of its focus on enhancing practice and expanding knowledge and understanding of the process of change, the co-operative inquiry group was deemed the most appropriate method to address the sociocultural barriers to supportive supervision (18).

## Methods

### Study design

**Fig. 1:**
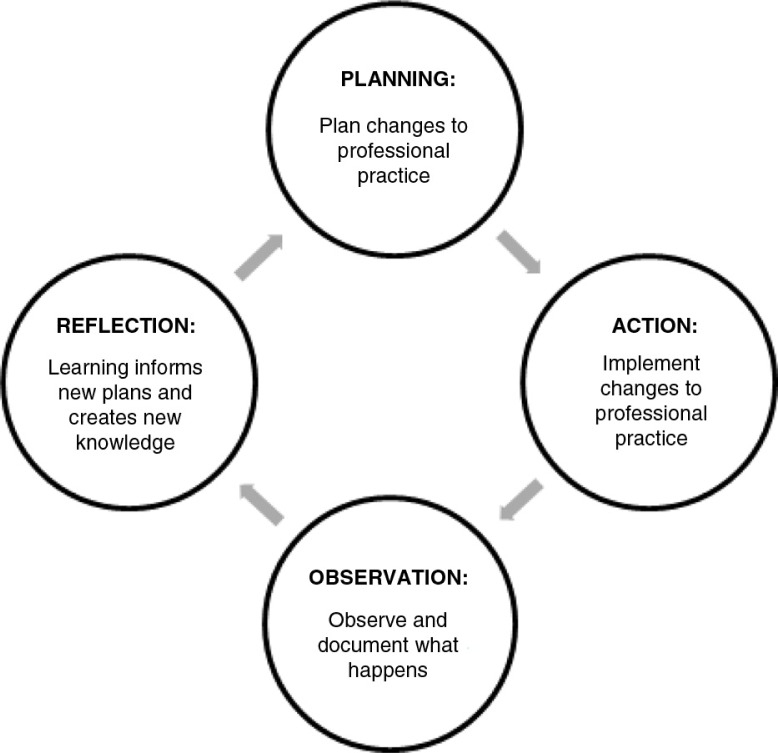
The co-operative inquiry action–reflection cycle (18, 19).

The co-operative inquiry group (CIG) is a form of participatory action research that involves a cyclic process of planning, action, observation, and reflection (Fig. 1) (18–21). The CIG method is a way of ‘working with professionals who would like to reflect on and change their practice’ and of ‘learning and reflection … [on] pertinent everyday practice related problems and attempting to resolve them as part of the research process’ (18, 21).

### The setting

The Ngamiland health district (population: 74,792), which has 29 primary healthcare facilities (clinics and health posts), is in the extreme north-western part of Botswana and is both rural and remote. The district has many areas with very difficult terrain which are inaccessible, especially during the rainy season. The headquarters of the district is in Maun, where the DHMT and cluster heads are based.

**Fig. 2:**
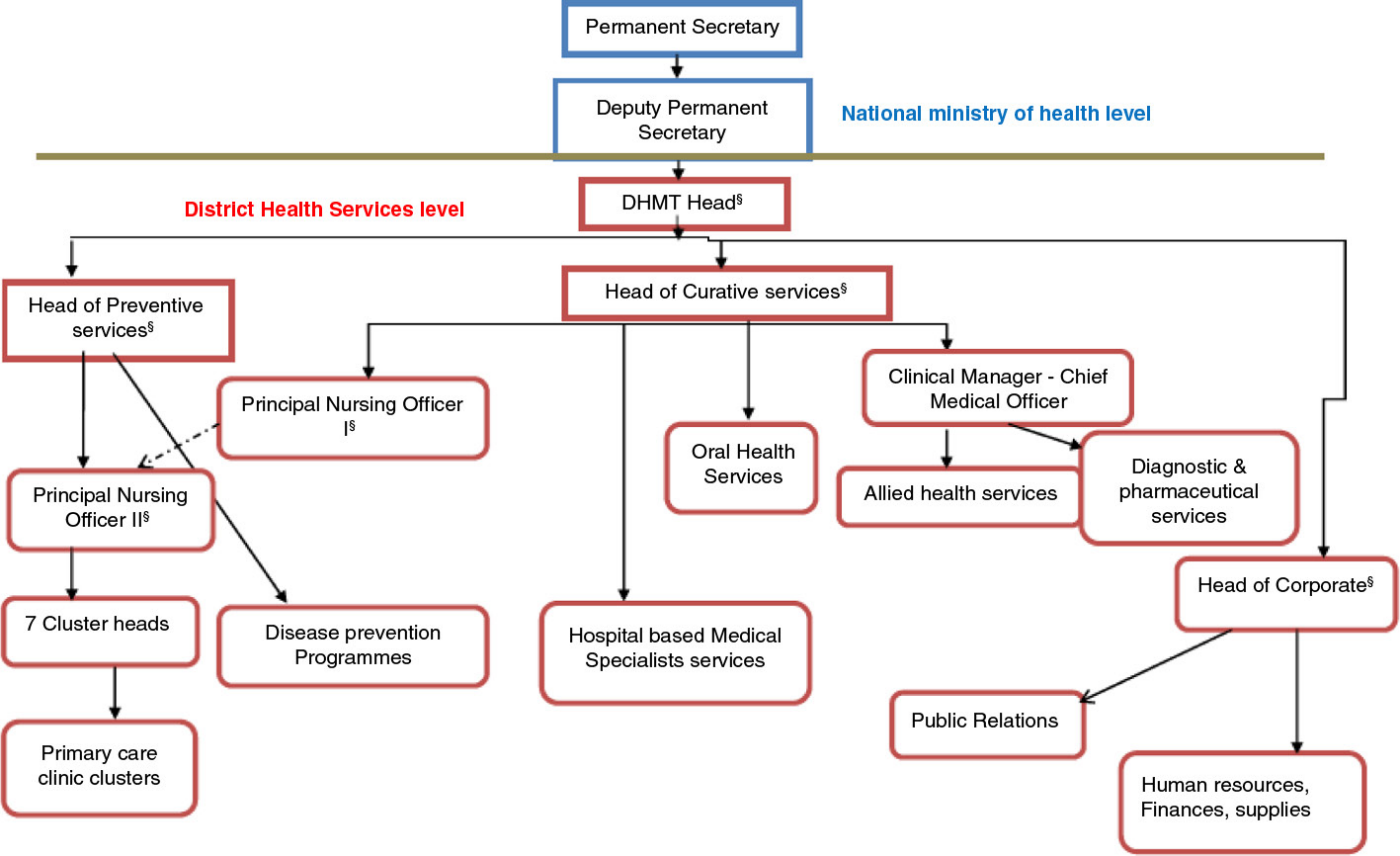
The organogram of the Ngamiland District health services. ^§^Member of the DHMT.

The Ngamiland DHMT was headed by a public health specialist, who was responsible for the overall leadership of the district health services. The head of preventive services, a senior nursing officer, had oversight of the primary healthcare and public health services. The head of curative services, a doctor, was mainly responsible for hospital-based clinical care. The head of corporate services, an administrator, was in charge of all the support services. The one principal nursing officer was mainly accountable for hospital-based nursing services while the subordinate principal nursing officer was the direct supervisor of the cluster heads (Fig. 2). The primary care clinics and health posts were functionally organised into seven clusters, each feeding into a larger ‘mother’ clinic. Each cluster was managed by a cluster head who was a nursing officer based at the larger clinic in the cluster. These were the direct supervisors of the healthcare workers in the clinics within their clusters.

At the time of this research, there were no guidelines for supervision of primary healthcare workers. However, all government employees, including primary healthcare workers, are mandated to sign an annual performance contract with their supervisors. The supervisors are therefore expected to meet at least quarterly with their subordinates to review progress and address challenges, but these meetings rarely took place (12). The interaction between the supervisors and their subordinates was largely limited to the submission of mandatory statistical reports, passing on directives from the National Ministry of Health and crisis management when problems arose (12, 13). Both the DHMT and cluster heads had scheduled regular meetings: monthly for the DHMT and weekly for the cluster heads.

### The CIG process

Members of the DHMT and cluster heads were invited to a meeting in Maun where the findings of prior research to assess the deficit in human resources for primary health in Botswana were presented. One of the important findings of the research was that there was inadequate support and supervision of primary healthcare workers, which had led to poor motivation, high attrition, and poor patient care (12). They were invited to participate in a co-operative inquiry that would address the question ‘How can managers change their practice to create a more supportive environment for primary care providers?’ Although they were initially sceptical about the validity of the CIG as a research method, all the managers consented to commit a year to participating in the study. Each group member was given a journal to record their actions and reflections. The group was trained to reflect using the adapted reflectivity 5-step method (21) (Table 1), as well as to give and receive feedback (22).

**Table 1:** Adapted reflectivity-5-step (19)

1. Read and re-read your observation and *observe your reaction, thoughts, and emotions, while doing justice to the text. Write them down one by one*
2. What main issue jumps out for you?
3. Formulate a theme from/on the basis of the issue.
4. Scrutinise your relationship with the theme. Look at whether this theme occurs often in your life, your work? Is this part of your pet theory? Do you want to re-formulate your theme here?
5. Select a theoretical concept (or lens) to give new meaning and understanding to the theme
6. Continue this process until the text is exhausted

The CIG meetings were conducted in quiet venues where refreshments were served, but there were no financial incentives for participation. The discussions were conducted in English, the official language of organisational transaction. The CIG went through three cycles between March 2013 and March 2014, each lasting 2–6 months (Table 2). ON facilitated three while RM facilitated two of the CIG meetings. At the meetings, each group member presented their personal actions, reflections, and new knowledge by responding to these questions: What did I do? What happened? What was different from what I expected? What did I not do? What did I do instead? What have I learnt from this? What is the issue now? What specific action should I take? (22). Other members of the group then gave feedback to the presenter. After individual presentations, the group reflected on overall experiences and what they had learnt as a group. They then planned individual and collaborative actions for the subsequent cycle (18, 22, 23). The facilitator reminded the group of the research question at each meeting to ensure alignment of actions and reflections with the purpose of the inquiry.

**Table 2:** Summary of the facilitated co-operative inquiry group meetings

Date	Type of meeting	Members of co-operative inquiry group in attendance
28/02/13	Introductory meeting	12
17/04/2013	Planning meeting	9
03/07/13	Co-operative inquiry group meeting	12
11/09/13	Co-operative inquiry group meeting	6
15–16/03/14	Building consensus of learning	9

At the meeting on 11 September 2013, in addition to the usual feedback, the results of a survey on the perceived current and desired organisational culture in Ngamiland health district were presented to the group. The results of the survey have been fully described elsewhere (13). The cultural values survey gave insight into how the district health service was experienced by the primary healthcare workers and what kind of organisational and leadership transformation would create a more enabling and supportive environment.

### Construction of new knowledge

The individual contributions by group members and the group discussions were audio recorded using digital voice recorders and then transcribed verbatim. After each meeting, ON summarised the transcripts and circulated, via email, the synopsis of the individual feedback and reflections, group's learning, and further plans for action during the subsequent cycle. Key insights and learning from the group's reflections during the CIG process are presented in the results section with quotations extracted from the transcripts to support and illustrate the learning in the words of the CIG members (18, 22).

The final meeting of the CIG group in March 2014 constructed a consensus of the group's learning using the nominal group technique to identify and prioritise the key issues (24, 25). Each member of the CIG was given an A4 sheet of paper and asked to quietly write their responses to the question: How can managers change their practice to create a more supportive environment for primary care providers? After about 15 min, the group came together and each member in turn gave one answer to the question until all the answers were recorded on a flip chart. The group then clarified the items and those that overlapped or were duplicates were combined. Each member was then given a voting paper and asked to write down five items that were the most important for them and score them from five (most important) to one (least important). The scores were then summated and the items ranked from what the CIG considered most to least important in terms of their learning.

## Ethical consideration

Approval to conduct the study was given by the University of Botswana Institutional Review Board, the Ngamiland district Ethics Committee, the Stellenbosch University Health Research Ethics Committee: Protocol Number: S13/03/051 and the Botswana Ministry of Health, Health Research Development Committee: Reference Number: PPME 13/18/1 V11 (368). Written consent was obtained from each study participant.

## Results

All the 12 (100%) district health services managers, which included five DHMT members and seven cluster heads, participated in the CIG. Two of the members were male doctors, one was a female administrator, and nine were female nurses. Two mangers left the district during the 12 months of the inquiry, and one was replaced by a new member who also joined the inquiry group.

The key actions carried out by the members of the CIG included developing their supervisory visit schedules and sharing them with their subordinates, conducting supervisory visits, addressing the difficulties identified by the supervisees during the visits and optimising the use of transport. The managers also adapted a supervisory checklist to use during their visits.

### Key insights and learning

Important lessons and key insights emerging from the CIG are discussed below.

#### Current supervision practice

Managers recognised that they had not had a culture of regular supervisory visits to their supervisees in the workplace and had tried to supervise remotely from their offices in Maun. They also realised that their current supervisory style was top-down and was characterised by demanding information and giving directions:

… like the analysis has shown that there is a lot of giving directions [making demands] and at times we just give directions not knowing the other people's side … what they want or what they would like to happen …. (Cluster head 1) [15/03/14]

#### Perceptions of healthcare workers’ experiences

The managers reported that the healthcare workers appreciated the new supervisory visits:

… if you visit them they really show they appreciate … they will just be telling you … even if things are so difficult … [it] shows that you care. (Cluster head 2) [03/07/13]

The managers on the contrary gained new insights into the challenges faced by the healthcare workers, for example, the long hours that many are expected to work:

… So we were still seeing patients at that late hour … I have learned that those guys [healthcare workers] are really working hard. They are trying their level best …. (DHMT member 1) [03/07/13]

One of the DHMT members visited some of the remote clinics for the first time and was shocked by the deplorable working and living conditions of the healthcare workers:

… I have been taking reports from the nurses maybe by phone … I never visited these facilities. … I [recently] visited three remote facilities and I realized that their living conditions … are very, very, very poor …. (DHMT member 2) [11/09/13]

#### Appreciation of the need for change in the supervisory paradigm

The managers realised that they needed to change their management style to include the more ‘human’ elements of supervision, and each manager selected one or more values to transform their supervisory style. The selected values were teamwork, information sharing, transparency, affirmation, caring, staff health, and collaboration. Each manager defined their understanding of the selected values as outlined below.

The values of transparency and information sharing encompassed making the organisational rules and regulations known to employees as well as sharing any pertinent information affecting their work:

… at the moment people discover what the rules are when they have transgressed them … [we should] be more transparent about the expectations …. (DHMT member 2) [15/03/14]

The values of caring and looking after staff health meant trying to create an environment that encouraged healthcare workers to be open with their supervisor about their stress and health issues:

… I am personally looking at caring … we need to really care about them and this will look at things like their health … so that even if they have problems they can freely come to me …. (Cluster head 3) [15/03/14]

The new caring supervision style encouraged healthcare workers to share personal problems which were then addressed:

… a lot of our staff members … were bringing sick leaves … we found out that they didn't have any money … they were living above their means. … didn't have money even for transport … they ended up going to get a fake sick leave … then it was agreed by the management that there should be some financial training … So this workshop was conducted last month for each and every person …. (Cluster head 5) [15/03/14]

The value of affirmation entailed acknowledgement, appreciation, and recognition of performance and excellence:

… when you work with people you should always appreciate what they do … So I visited all of my facilities and during our interactions I appreciated what they are doing so that this can motivate them …. (Cluster head 2) [15/03/14]

The value of collaboration meant shared decision making and problem solving as opposed to all decisions and plans being made by the supervisors:

…we will sit down and make decisions together so that … everybody has an input on what is supposed to happen …. (Cluster head 1) [15/03/14]

The value of teamwork emphasised the importance of each employee to the success of the whole team:

… teamwork, I was looking at people … regardless of their rank in the organisation to look at themselves as being important in the organisation …. (DHMT member 3) [15/03/14]

#### Barriers to supportive supervision and efforts to overcome them

Hindrances to supportive supervision included inadequate supervisory skills, lack of transport, and lack of support from the National Ministry of Health. A number of managers felt inadequately prepared to deal with difficult and uncooperative subordinates:

… I have realized that even in one mother clinic the nurses there they are having problems with communication … Now I was just confused I didn't know what to do …. (Cluster head 5) [11/09/13]

… I had a problem in my cluster because of drivers not cooperating. I had to call all of them but unfortunately one of them was a bit problematic … he didn't want to cooperate …. I've learnt that a supervisor should have a skill of leading …. (Cluster head 7) [03/07/13]

The other big challenge was unscheduled meetings, workshops, and visits by the National Ministry of Health, which significantly interfered with the managers’ ability to effectively implement their own plans to the point where they sometimes felt defeated:

… I feel the sense … [of] being defeated … if you are told that the permanent secretary is coming … you cannot say ‘no’ or leave it to someone else …. (DHMT member 3) [03/07/13]

Shortage of transport was one of the major challenges faced by the primary healthcare workers and supervisors and consequently managers were sometimes stranded in remote areas when conducting scheduled supervisory visits:

… I had to stay in Toteng to wait for any transport from Maun to Sehithwa or that other side … it's really a very big challenge because I scheduled that [supervision visit] but still with their transport [shortage] it was difficult …. (DHMT member 1) [03/07/13]

The managers realised though that the shortage of transport was exacerbated by a failure to utilise the existing vehicles and drivers optimally to benefit all the clinics:

… When the one clinic did not have a vehicle, drivers from clinics with vehicles just passed it without going to check if there was anything they needed transported to Maun …. (Cluster head 7) [03/07/13]

The managers also recognised that there were opportunities to piggyback their supervisory visits onto other activities in order to ameliorate the transport challenge:

… I realized that the anti-Retroviral [programme] has regular transport … there is also space for me to go for supervisory visits …. (Cluster head 3) [11/09/13]

The managers also started collaborating amongst themselves to solve complicated transport challenges:

… one of the clinics in my cluster [operates for] 24 hours … it has been running with three drivers … none of them were knocking off … so I … called other cluster heads [as] … some of their drivers were just not doing anything because they had no vehicles … and two drivers … came to join my drivers …. (Cluster head 7) [03/07/13]

#### Appreciation of professional growth afforded by the CIG process

The managers appreciated the new skills of reflection, giving and receiving feedback and listening actively, which they viewed as important to their personal development as leaders and which were different from the usual theoretical workshops. They also discovered their ability to influence some of the challenges that plagued them:

… I think … I have to be very honest with the people above [Ministry of Health management] and give them the real picture. Because when … I agree to all their schedules … it's like it is fine …. (DHMT member 8) [03/07/13]

### Ownership of the transformation process

Although initially the CIG members referred to the CIG as ‘your research project’, once they appreciated its impact they embraced it as theirs:

… my project now … it's better I shift things now to … look into all facilities one by one and … improve their conditions of living, especially in the issue of maintenance …. (DHMT member 2) [11/09/13]

By 16 March 2014, the CIG members were confident they could implement supportive supervision without external facilitation as they had already started to integrate it into their internal processes.

### Consensus on the findings of the CIG

Members of the inquiry group collectively contributed 20 items they considered key to supportive supervision in terms of their learning (Table 3). Availability of resources and training of managers were considered the most important enablers of supportive supervision.

**Table 3:** Consensus on how to strengthen supportive supervisory practice of district health managers

Priority level	How can managers change their practice to create a more supportive environment for primary care providers
1	Provide the resources needed for supervision, especially transport Empower DHMT to have skills in supervision, like problem solving
2	Improve sharing of information from DHMT and cluster heads to health workers
3	Transform the organisational culture (values) to bring more congruence between actual experience and espoused/desired values
4	Offer psychosocial support to staff/be aware what is available to improve staff wellness
5	Create space during the supervisory visit to meet with staff and hear what is happening and do collaborative problem solving Give feedback to MOH on the reality of the situation on the ground such as staffing levels and effects of transfer policy on staff motivation
6	Have norms and standards of what should be in place in terms of equipment, medicines, and human resources according to facility level Have an appreciative style of supervision (not the blame game)
7	Change the leadership style to be more of a collaborative practice (teamwork) and model this downward
8	Clarify roles and contribution to supervision of primary healthcare workers by different levels of managers Improve coordination between managers/supervisors on how they supervise Have a policy on the length of stay in rural areas Build resilience of leaders by offering them support. e.g., psychological/emotional
9	Increase awareness of the importance of supervisory visits in DHMT Respond to the deficiencies in resources at the level of clinics/health posts Monitor and evaluate to hold people accountable for progress with actions/responsibilities
10	Give constant feedback to the healthcare workers on progress of jointly identified challenges and strategies Benchmark ourselves against best practice in supervision Recognise leaders/managers through promotion

## Discussion

This study has demonstrated that developing supportive supervision for primary care providers in Ngamiland required significant leadership and contextual transformation. The managerial leadership of the district gained insight into the need for personal development and to build more effective communication and relationships between multiple role players. In addition, it became clear that major contextual changes were required to provide an environment within which supportive supervision could flourish. The most essential changes within the context included the need for transportation and a culture of visiting the healthcare workers, even in remote rural settings.

District health managers recognised the need to change their supervisory paradigm from authoritarian to supportive supervision (14). The participatory process enabled them, with assistance from colleagues, to challenge their assumptions and change their practice (20, 21). The change in paradigm was a necessary step for implementing supportive supervision, which requires motivation, investment, and shift in behaviour (14). Moreover, dictatorial supervision demotivates healthcare workers, while supportive supervision has been shown, although inconsistently, to improve health workforce performance, productivity, quality of care, motivation, and retention (16, 26–28).

Although the managers in Ngamiland health district were inspired to be supportive supervisors, motivation can only be sustained if identified obstacles are addressed and supervision is prioritised. The Ministry of Health has an important role to develop strategies that support supervisors and improve their performance (29, 30). Despite formidable challenges, the managers made significant inroads in solving some of them and were optimistic that with adequate support, some degree of autonomy, and sufficient resources they could resolve many more.

The ideas expressed in the CIG's consensus of learning correlate very closely with the organisational values that the primary healthcare workers in Ngamiland and Mahalapye health districts would like to experience in the district health services. These sought after values were transparency, professional growth, staff recognition, shared decision making, accountability, productivity, leadership development, and teamwork (13). Similar organisational values were also called for by primary healthcare workers in Cape Town, suggesting that these challenges may be widely prevalent in district health services in the region (31).

Locally developed evidence on how to create more supportive supervision is more likely to be seen as relevant, applicable, and feasible to implement by policymakers in Botswana and the region (14, 15). It is notable, however, that the consensus contains many of the tenets of supportive supervision that are recognised internationally, such as a shift from checklists to proactive planning, collaborative problem solving, promoting of high standards, teamwork, and two-way communication (14, 30, 31). In addition, the learning of the CIG recognised that supportive supervision should be prioritised at all levels of the healthcare system, and that it requires adequate resources and skilled supervisors, factors which are known to be integral to successful supportive supervision (14, 30). Despite scheduling challenges, flexibility, excellent rapport, and empathetic communication facilitated value-based reflectivity with busy district managers in a resource-limited setting. The CIG approach therefore offers a potential vehicle for the transformative process necessary to make the well-known tenets of supportive supervision a reality in district health services.

The CIG process remained well aligned with its purpose, and the members gradually developed a sense of ownership of the inquiry process. The range of possible actions by the CIG members was significantly limited by the context, particularly the lack of transport. Reflectivity developed well during the inquiry as demonstrated by the richness of the final consensus. Group meetings were difficult to organise due to the busy schedules of the management team, and more regular meetings might have enabled the inquiry to progress faster and to go deeper.

The consensus on supportive supervision was developed in one of Botswana's 28 health districts which may limit its applicability nationally. This, however, is unlikely as previous studies by the team have shown that the primary health workforce in Botswana have almost identical experiences of the district health services (12, 13). The consensus is also congruent with what is known to be important for supportive supervision and may thus be transferrable to wider settings (14, 29, 30, 32).

There is a risk of desirability and confirmation biases, especially because of the hierarchical composition of the group, and some participants may have deferred to the learning of more senior members. However, the democratic nature of the CIG with emphasis on individual action, reflection, and learning significantly reduces these sources of bias (18).

This study was part of a quasi-experimental research with pre- and post-motivation and organisational culture surveys, and the results of the surveys are still to be published. However, further research is still required on the healthcare workers’ perceptions of the changes and how these can be sustained.

## Conclusion

Through participatory action research, district health managers in the Ngamiland health district have come to appreciate the value of supportive supervision and have changed their management style. The managers also became aware of the experiences of the primary healthcare workers in their district and the challenges to implementing supportive supervision. Furthermore, the managers developed a consensus on what is required to develop supportive supervision that can potentially be integrated into the management of district health services across the country and may be applicable in other similar settings. Supportive supervision should be prioritised at all levels of the health system and be adequately resourced and accompanied by training and mentoring of the managers.

## Paper context

Supportive supervision is associated with higher levels of health workers’ motivation, performance, retention, and patient outcome. The bulk of this evidence, however, is derived from high-income countries. Nevertheless, this paper reports that, through a participatory process, district health managers in resource-limited settings can become supportive supervisors. In addition, for supportive supervision to flourish, the supervisors must possess the technical skills and appropriate attitudes and be supported by an enabling policy and resource environment.

## References

[1] World Health Organization (2008). Primary care: now more than ever, 2008 World Health Report.

[2] Nkomazana O, Peersman W, Willcox M, Mash R, Phaladze N (2014). Human resource for health in Botswana: a situational analysis. Afr J Prm Health Care Fam Med.

[3] World Health Organization (2006). The World Health Report 2006 – working together for health.

[4] Moosa S, Wojczewski S, Hoffmann K, Poppe A, Nkomazana O, Peersman W (2014). The inverse primary care law in sub-Saharan Africa: a qualitative study of the views of migrant health workers. Br J Gen Pract.

[5] Sousa A, Scheffler RM, Koyi G, Ngah SN, Abu-Agla A, M'kiambati HM (2014). Health labour market policies in support of universal health coverage: a comprehensive analysis in four African countries. Hum Resour Health.

[6] World Health Organization (2010). WHO global code of practice on the international recruitment of health personnel.

[7] Dolea C, Stormont L, Braichet J-M (2010). Evaluated strategies to increase attraction and retention of health workers in remote and rural areas. Bull World Health Organ.

[8] Lehmann U, Dieleman M, Martineau T (2008). Staffing remote rural areas in middle- and low-income countries: a literature review of attraction and retention. BMC Health Serv Res.

[9] Gow J, George G, Mwamba S, Ingombe L, Mutinta G (2013). An evaluation of the effectiveness of the Zambian Health Worker Retention Scheme (ZHWRS) for rural areas. Afr Health Sci.

[10] 
Antwi J, Phillips DC (2013). Wages and health worker retention: evidence from public sector wage reforms in Ghana. J Dev Econ.

[11] Buykx P, Humphreys J, Wakerman J, Pashen D (2010). Systematic review of effective retention incentives for health workers in rural and remote areas: towards evidence-based policy. Aust J Rural Health.

[12] Nkomazana O, Mash R, Shaibu S, Phaladze N (2015). Stakeholders’ perceptions on shortage of healthcare workers in primary healthcare in Botswana: focus group discussions. PLoS One.

[13] Nkomazana O, Mash R, Phaladze N (2015). Understanding the organisational culture of two health districts in Botswana: implications for retention of primary healthcare workers. Afr J Prm Health Care Fam Med.

[14] Marquez L, Keane L (2002). Making supervision supportive and sustainable: new approaches to old problems.

[15] Moran AM, Coyle J, Pope R, Boxall D, Nancarrow SA, Young J (2014). Supervision, support and mentoring interventions for health practitioners in rural and remote contexts: an integrative review and thematic synthesis of the literature to identify mechanisms for successful outcomes. Hum Resour Health.

[16] Bailey C, Blake C, Shriver M, Cubaka VK, Thomas T, Martin Hilber A (2016). A systematic review of supportive supervision as a strategy to improve primary healthcare services in Sub-Saharan Africa. Int J Gynaecol Obstet.

[17] Clements CJ, Streefland PH, Malau C (2007). Supervision in primary health care – can it be carried out effectively in developing countries?. Curr Drug Saf.

[18] Malterud K (1995). Action research – a strategy for evaluation of medical interventions. Fam Pract.

[19] Mash B, Meulenberg-Buskens I (2001). ‘Holding it lightly’: the co-operative inquiry group: a method for developing educational materials. Med Educ.

[20] Denscombe M (2010). The good research guide: for small-scale social research projects.

[21] Mash B (2002). The development of distance education for general practitioners on common mental disorders through participatory action research.

[22] McGill I, Brockbank A (2004). The action learning handbook: powerful techniques for education, professional development and training.

[23] Meyer J (2000). Evaluating action research. Age Ageing.

[24] Lloyd-Jones G, Fowell S, Bligh JG (1999). The use of the nominal group technique as an evaluative tool in medical undergraduate education. Med Educ.

[25] Reid S, Mash B, Blitz-Lindeque J (2006). How to prioritise a community-based intervention. South African Family practice manual.

[26] Roberton T, Applegate J, Lefevre AE, Mosha I, Cooper CM, Silverman M (2015). Initial experiences and innovations in supervising community health workers for maternal, newborn, and child health in Morogoro region, Tanzania. Hum Resour Health.

[27] Bosch-Capblanch X, Liaqat S, Garner P (2011). Managerial supervision to improve primary health care in low- and middle-income countries. Cochrane Database Syst Rev.

[28] Bradley S, Kamwendo F, Masanja H, Waxman R, Boostrom C, McAuliffe E (2013). District health managers’ perceptions of supervision in Malawi and Tanzania. Hum Resour Health.

[29] Program for Appropriate Technology in Health (2003). Guidelines for implementing supportive supervision: a step-by-step guide with tools to support immunization.

[30] Rowe AK, de Savigny D, Lanata CF, Victora CG (2005). How can we achieve and maintain high-quality performance of health workers in low-resource settings?. Lancet.

[31] Mash R, Govender S, Isaacs AA, De Sa A, Isaacs A (2013). An assessment of organisational values, culture and performance in Cape Town's primary care services. S Afr Fam Pract.

[32] Frimpong J, Helleringer S, Awoonor-Williams JK, Yeji F, Phillips JF (2011). Does supervision improve health worker productivity? Evidence from the Upper East Region of Ghana. Trop Med Int Health.

